# Clinico-Radiological Profile of Patients With Lateral Medullary Syndrome: A Five Years Observation From a Single-Centered Tertiary Hospital in Nepal

**DOI:** 10.7759/cureus.28834

**Published:** 2022-09-06

**Authors:** Ramesh Shrestha, Rohit Pandit, Ankit Acharya, Ghanshyam Kharel, Anzilmani S Maharjan, Subash Phuyal, Suresh Bishokarma

**Affiliations:** 1 Cardiology, The Heart and Vascular Institute of Michigan, Detroit, USA; 2 Neurology, Upendra Devkota Memorial (UDM) National Institute of Neurological and Allied Sciences, Kathmandu, NPL; 3 Cardiology, The Heart and Vascular Institute, Michigan, USA; 4 Internal Medicine, Om Saibaba Memorial Hospital, Kathmandu, NPL; 5 Department of Public Health, Nobel College, Pokhara University, Kathmandu, NPL; 6 Central Department of Public Health (CDPH), Institute of Medicine, Tribhuvan University, Kathmandu, NPL; 7 Neurology, Upendra Devkota Memorial (UDM) National Institute of Neurological and Allied Sciences, Kathamandu, NPL; 8 Neuroimaging and Interventional Neuroradiology, Upendra Devkota Memorial (UDM) National Institute of Neurological and Allied Sciences, Kathmandu, NPL; 9 Neurosurgery, Upendra Devkota Memorial (UDM) National Institute of Neurological and Allied Sciences, Kathmandu, NPL

**Keywords:** vertebral artery, pica, stroke, radiologic subtypes, rostrocaudal, mri, lateral medullary syndrome

## Abstract

Objective: We aim to correlate the prevalence of symptoms of the lateral medullary syndrome (LMS) based on radiological classification.

Methods: A five-year record of 41 patients diagnosed with LMS and admitted to a tertiary care center in Nepal was reviewed. We used chi-square tests to compare symptoms between rostral and caudal groups and different horizontal subtypes.

Results: The subtype prevalence in the horizontal classification of LMS was large (31.7%), lateral (22%), dorsal (19.5%), typical (14.6%), and ventral (12.2%). The most common symptoms in the typical subtype of the horizontal classification were: pain/temperature loss in the contralateral body (7.3%) and dysphagia (7.3%); in the ventral subtype, swaying on the Romberg test (12.2%), dysarthria (9.8%) and dizziness (9.8%); in the dorsal subtype, headache (12.2%) and vomiting (12.2%). Whereas headache (22.2%) and lateropulsion on standing (14.6%), swaying on the Romberg test (14.6%), nausea/vomiting (14.6%) were common in the large subtype, and nausea/vomiting (19.5%) and headache (17.1%) in the lateral subtypes. Whereas, in rostrocaudal classification, the rostral subtype (61%) was more common than the caudal subtype (31%). There was no significant variation in symptoms based on the rostrocaudal classification of LMS.

Conclusion: The common clinical manifestations are different for different radiological subtypes of LMS. Further comprehensive studies are essential to understand the prevalence of symptoms in different radiological subtypes and the clinical-radiologic correlation in LMS.

## Introduction

Lateral medullary syndrome (LMS) is a clinical syndrome caused by ischemic infarction of the lateral medulla. This is most commonly caused by obstruction of the intracranial portion of the vertebral artery, followed by the posterior inferior cerebellar artery (PICA) and its branches [1-3]. Patients present with a variety of symptoms, including crossed sensory deficit (ipsilateral face, contralateral body), ipsilateral Horner syndrome, cerebellar ataxia, vertigo, nystagmus, dysphagia, dysarthria, hoarseness, ipsilateral diminished gag reflex, skew deviation of eyes, headache, and nausea/vomiting [4]. Radiologically, in the transverse section, the lateral medullary syndrome is classified as typical, ventral, large, lateral, and dorsal, whereas in the sagittal section, there are rostral and caudal infarctions [2]. Limited data are available regarding the clinical and radiological correlation of LMS [2,4]. Our study aims to correlate the prevalence of symptoms of LMS based on radiological classification.

## Materials and methods

A descriptive cross-sectional study was conducted on hospital records of patients admitted and treated with the diagnosis of LMS over five years between 2017 and 2022 A.D. at Upendra Devkota Memorial (UDM) National Institute of Neurological and Allied Sciences, a tertiary care hospital in Kathmandu, Nepal. Ethical approval was granted (Reference No. 120/2022) by the institutional review board of the UDM National Institute of Neurological and Allied Sciences.

The demographic characteristics of the participants, including age and sex; clinical characteristics, including risk factors, signs, and symptoms of LMS (standard hospital emergency/inpatient history taking, neurological examination form was used for LMS); and radiological findings were retrospectively retrieved from the hospital registers. All 41 patients underwent an MRI brain stroke protocol. All patients underwent brain scanning using a 1.5T MRI scanner in a horizontal plane at 3 mm intervals. The gadolinium-enhanced T2-weighted axial and sagittal T1-weighted images, as well as the MRA imaging, were retrospectively assessed and classified by one neurologist and one neurointerventionalist who were both blinded to the clinical information.

The study included patients who had diagnostic MRI findings and at least two of the typical LMS symptoms. The typical symptoms and signs include vertigo, nausea/vomiting, gait ataxia, Horner's sign, dysphagia, hoarseness, and any spinothalamic sensory symptoms and signs.

We excluded patients with multiple anatomical infarctions that involved more than just the lateral medulla, non-significant MRI lesions, MRIs performed more than 171 hours after the onset of symptoms, and initial MRI-negative patients.

The lesions were divided rostrocaudally into the caudal medulla, which had a relatively rounded shape without lateral surface bulging, and the rostral medulla, which had a significant dorsolateral bulging caused by the restiform body [5]. Horizontal classification on MRI was considered equivalent to the anatomically transverse section.

**Figure 1:**
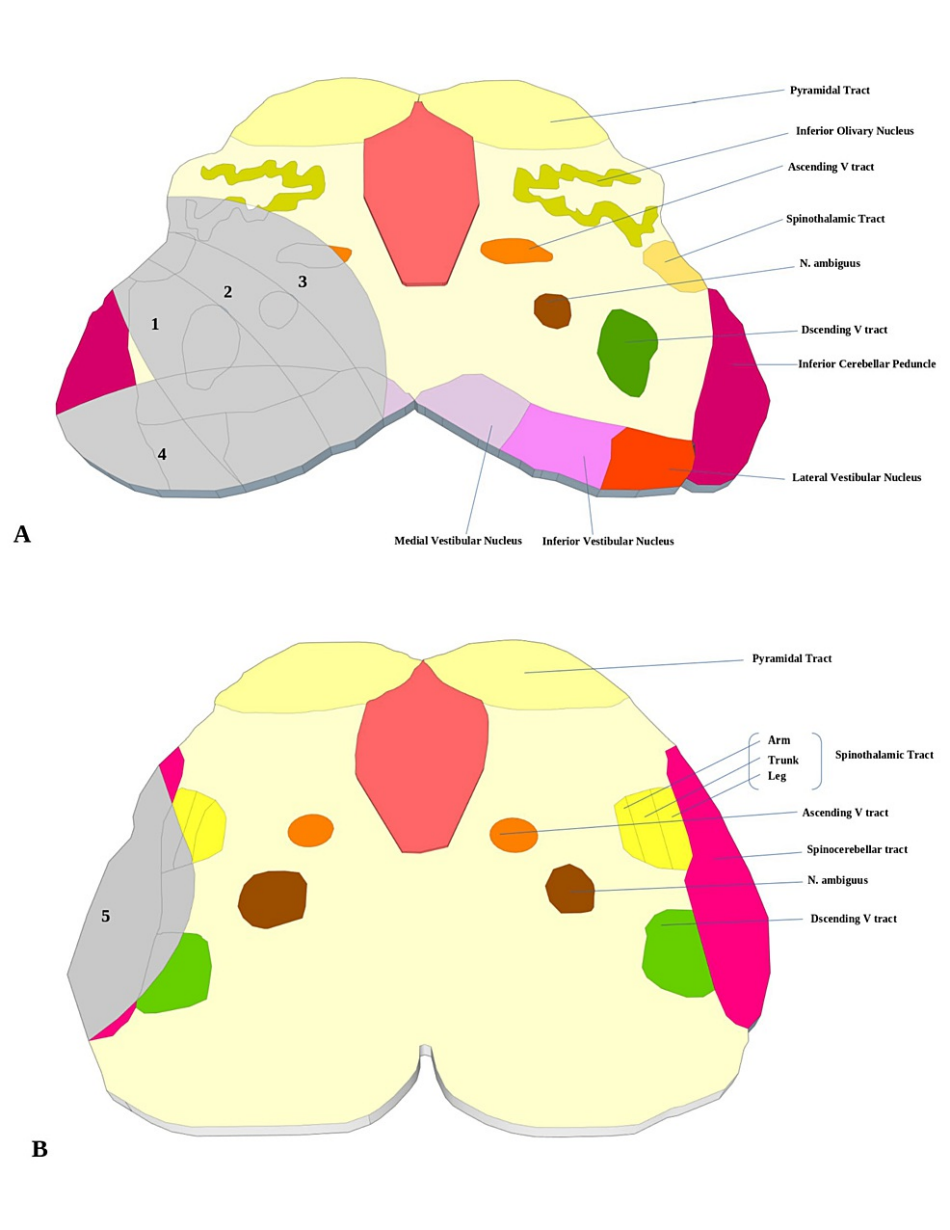
Medullary structures at the rostral (A) and caudal levels are depicted in this diagram (B) Shaded regions reflect various patterns of lateral medullary infarction. 1 + 2 = typical; 2 + 3 = ventral; 1 + 2 + 3 = large; 4 = dorsal; 5 = lateral. Adapted from Kim JS. Pure lateral medullary infarction: Pure lateral medullary infarction: clinical-radiological correlation of 130 acute, consecutive patients. Brain. 2003 Aug;126(Pt 8):1864-72 [2]. The Permission was obtained from the original Oxford Academic publisher(License no. 5367780441386) DOI: 10.1093/brain/awg169.

Horizontally, subtypes are defined as follows (Figure 1). (i) Typical type (1+2): sparing the most dorsolateral area, diagonal band-shaped lesions. (ii) Ventral type (2+3): lesions located more ventrally and affect the inferior limb were largely spared. (iii) Large type (1+2+3): lesions that extend dorsally to affect the majority of the dorsolateral region and ventrally to affect a portion of the olivary nucleus. (iv) Dorsal type (4): lesions that are confined to the dorsolateral or most dorsal region. (v) Lateral type (5): the superficial region of the lateral caudal medulla without extending dorsally [2,5].

The large arteries are the extracranial cerebral arteries (carotid and vertebral arteries) and intracranial muscular arteries coursing through the subarachnoid space [6]. The small vessels are the deep perforating arteries and superficial perforating arteries that penetrate the brain tissue [6].

Data entry and all statistical tests were performed on IBM SPSS Version 25 (SPSS Inc., Chicago, IL). A chi-square test was used to compare the symptoms of LMS between rostral and caudal groups and different horizontal subtypes. P-values of <0.05 were regarded as indicating significance.

## Results

A total of 41 patients diagnosed and admitted with LMS were reviewed for the study, of which 7 (17.1%) were female and 34 (82.9%) were male. The population in the study ranged from 25 to 86 years old, with a mean age of 53.95 ±15.13 years.

The most common vestibulocerebellar symptoms were nausea/vomiting 23 (56.1%), swaying 22 (53.7%), dizziness 17 (41.15%), and diplopia 9 (22.2%); bulbar symptoms were dysarthria 10 (24.4%) and dysphagia 9 (22%); autonomic symptoms included Horner's syndrome 4 (9.8%) and hiccups 2 (4.9%); sensory symptoms included pain and temperature loss in the contralateral body 9 (22%) and ipsilateral face 7 (17.1%); and atypical symptoms included facial nerve palsy 7 (17.1%, hemiparesis 3 (7.3%) and trigeminal neuropathy in 2 (4.9%) patient (Table 1).

**Table 1:** Prevalence of symptoms/signs in patients diagnosed with lateral medullary syndrome (N=41)

Symptoms/signs	Frequency
Headache	24(58.5%)
Change in level of consciousness	6(14.6%)
Vestibulocerebellar	
Nausea/vomiting	23(56.1%)
Swaying	22(53.7%)
Dizziness	17(41.5%)
Lateropulsion	10(24.4%)
Diplopia	9(22.0%)
Nystagmus	7(17.1%)
Ataxia	7(17.1%)
Vertigo	4(9.8%)
Bulbar muscle involvement	
Dysarthria	10(24.4%)
Dysphagia	9(22.0%)
Loss of gag reflex	3(7.3%)
Hoarseness	2(4.9%)
Autonomic sign	
Horner’s syndrome	4(9.8%)
Hiccup	2(4.9%)
Sensory sign	
Loss of pain and temperature in the contralateral body	9(22.0%)
Loss of pain and temperature in the ipsilateral face	7(17.1%)
Atypical presentation	
Facial nerve palsy	7(17.1%)
Hemiparesis	3(7.3%)
Trigeminal neuropathy	2(4.9%)

Based on the MRI findings in the horizontal classification, a large infarction was seen in 13 (31.7%), lateral in 9 (22.0%), dorsal in 8 (19.5%), typical in 6 (14.6%), and ventral in 5 patients (12.2%), whereas, in a rostrocaudal distribution, rostral infarction was seen in 25 (61.0%), and caudal in 16 patients (39.0%). The large subtype was the most common rostrally, affecting 10 patients (24.4%). However, the lateral subtype was most prevalent caudally, with involvement in five patients (12.2%) (Table 2).

**Table 2:** Radiological classification (MRI based) of lateral medullary syndrome (N=41) (P-value = 0.282)

Rostro-caudal	Horizontal					
	Large	Lateral	Dorsal	Typical	Ventral	Total
Rostral	10(24.4%)	4(9.8%)	5(12.2%)	4(9.8%)	2(4.9%)	25(61.0%)
Caudal	3(7.3%)	5(12.2%)	3(7.3%)	2(4.9%)	3(7.3%)	16(39.0%)
Total	13(31.7%)	9(22.0%)	8(19.5%)	6(14.6%)	5(12.2%)	41(100%)

We attempted to divide the lesions into the transverse section (Table 3) and rostrocaudal section (Table 4), respectively, to highlight potential clinical differences.

**Table 3:** Neurological symptoms/signs and MRI horizontal classification (N=41)

Symptoms/signs	Typical (n=6)	Ventral (n=5)	Dorsal (n=8)	Large (n=13)	Lateral (n=9)	Total	P-Value
Headache	1(2.4%)	2(4.9%)	5(12.2%)	9(22.0%)	7(17.1%)	24(58.5%)	0.951
Change in level of consciousness	0(0.0%)	0(0.0%)	3(7.3%)	1(2.4%)	2(4.9%)	6(14.6%)	0.188
Vestibulocerebellar							
Nausea/vomiting	2(4.9%)	2(4.9%)	5(12.2%)	6(14.6%)	8(19.5%)	23(56.1%)	0.403
Swaying	3(7.3%)	5(12.2%)	2(4.9%)	6(14.6%)	6(14.6%)	22(53.7%)	0.653
Dizziness	2(4.9%)	4(9.8%)	4(9.8%)	4(9.8%)	3(7.3%)	17(41.5%)	0.364
Lateropulsion	1(2.4%)	0(0.0%)	2(4.9%)	6(14.6%)	1(2.4%)	10(24.4%)	0.099
Diplopia	2(4.9%)	0(0.0%)	2(4.9%)	3(7.3%)	2(4.9%)	9(22.0%)	0.618
Nystagmus	1(2.4%)	1(2.4%)	1(2.4%)	3(7.3%)	1(2.4%)	7(17.1%)	0.745
Ataxia	2(4.9%)	0(0.0%)	0(0.0%)	2(4.9%)	3(7.3%)	7(17.1%)	0.239
Vertigo	0(0.0%)	1(2.4%)	0(0.0%)	1(2.4%)	2(4.9%)	4(9.8%)	0.439
Bulbar muscle involvement							
Dysarthria	0(0.0%)	4(9.8%)	2(4.9%)	2(4.9%)	2(4.9%)	10(24.4%)	0.028
Dysphagia	3(7.3%)	2(4.9%)	1(2.4%)	2(4.9%)	1(2.4%)	9(22.0%)	0.28
Loss of gag reflex	1(2.4%)	1(2.4%)	1(2.4%)	0(0.0%)	0(0.0%)	3(7.3%)	0.404
Hoarseness	0(0.0%)	1(2.4%)	0(0.0%)	1(2.4%)	0(0.0%)	2(4.9%)	0.425
Autonomic sign							
Horner’s syndrome	0(0.0%)	1(2.4%)	1(2.4%)	1(2.4%)	1(2.4%)	4(9.8%)	0.845
Hiccup	0(0.0%)	0(0.0%)	0(0.0%)	1(2.4%)	1(2.4%)	2(4.9%)	0.745
Sensory sign							
Loss of pain and temperature in the contralateral body	3(7.3%)	0(0.0%)	1(2.4%)	5(12.2%)	0(0.0%)	9(22.0%)	0.057
Loss of pain and temperature at the ipsilateral face	2(4.9%)	1(2.4%)	0(0.0%)	3(7.3%)	1(2.4%)	7(17.1%)	0.5
Atypical presentation							
Facial palsy	1(2.4%)	0(0.0%)	2(4.9%)	4(9.8%)	0(0.0%)	7(17.1%)	0.291
Hemiparesis	0(0.0%)	1(2.4%)	1(2.4%)	1(2.4%)	0(0.0%)	3(7.3%)	0.610
Trigeminal neuropathy	0(0.0%)	1(2.4%)	0(0.0%)	1(2.4%)	0(0.0%)	2(4.9%)	0.425

The most common symptoms in horizontal classification were pain and temperature loss in the contralateral body, dysphagia, and swaying, with a prevalence of 7.3% in each typical subtype. In the ventral subtype, swaying was 5(12.2%), dysarthria 4(9.8%), and dizziness 4(9.8%). In the dorsal subtype, headache 5(12.2%), nausea/vomiting 5(12.2%), dizziness 4(9.8%), and lateropulsion on standing 2(4.9%). In the large subtype, headaches were 9(22%), nausea/vomiting 6(14.6%), swaying 6(14.6%), lateropulsion on standing 6(14.6%), and loss of pain and temperature in the contralateral body 5(12.2%). In lateral subtypes, nausea/vomiting 8(19.5%), headache 7(17.1%), swaying 6(14.6%).

**Table 4:** Neurological symptoms/signs and MRI rostro-caudal classification (N=41)

Symptoms/signs	Rostral(n=25)	Caudal(n=16)	Total	P-value
Headache	14(34.1%)	10(24.4%)	24(58.5%)	0.476
Change in level of consciousness	4(9.8%)	2(4.9%)	6(14.6%)	0.489
Vestibulocerebellar				
Nausea/Vomiting	14(34.1%)	9(22.0%)	23(56.1%)	0.078
Swaying	10(24.4%)	12(29.7%)	22(53.7%)	0.256
Dizziness	9(22.0%)	8(19.5%)	17(41.5%)	0.938
Lateropulsion	4(9.8%)	6(14.6%)	10(24.4%)	0.747
Diplopia	5(12.2%)	4(9.8%)	9(22.0%)	0.178
Nystagmus	3(7.3%)	4(9.8%)	7(17.1%)	0.529
Ataxia	4(9.8%)	3(7.3%)	7(17.1%)	0.839
Vertigo	2(4.9%)	2(4.9%)	4(9.8%)	0.877
Bulbar muscle involvement				
Dysarthria	4(9.8%)	6(14.6%)	10(24.4%)	0.319
Dysphagia	7(17.1%)	2(4.9%)	9(22.0%)	0.032
Loss of gag reflex	1(2.4%)	2(4.9%)	3(7.3%)	0.276
Hoarseness	1(2.4%)	1(2.4%)	2(4.9%)	0.915
Autonomic sign				
Horner’s syndrome	2(4.9%)	2(4.9%)	4(9.8%)	0.877
Hiccup	0(0.0%)	2(4.9%)	2(4.9%)	0.119
Sensory sign				
Loss of pain and temperature in the contralateral Body	8(19.5%)	1(2.4%)	9(22.0%)	0.016
Loss of pain temperature at the ipsilateral face	3(7.3%)	4(9.8%)	7(17.1%)	0.529
Atypical presentation				
Facial Palsy	5(12.2%)	2(4.9%)	7(17.1%)	0.301
Hemiparesis	2(4.9%)	1(2.4%)	3(7.3%)	0.639
Trigeminal neuropathy	1(2.4%)	1(2.4%)	2(4.9%)	0.915

In rostrocaudal classification, the rostral and caudal divisions displayed a wide range of symptoms. However, headache 14(34.1%) in rostral vs 10 (24.4%) in caudal, nausea/vomiting 14 (34.1%) vs 9 (22.0%), change in the level of consciousness 4(9.8%) vs 10 (24.4%), dysphagia 7(17.1%) vs 2(4.9%), loss of pain and temperature 8(19.5%) vs 1(2.4%), and facial nerve palsy 5(12.2%) vs 2(4.9%) are more common differences in rostral vs caudal classification. Caudally, swaying was 12(29.7%) vs 10(24.4%) rostrally, lateropulsion on standing, dysarthria, 6(14.6%) vs 4(9.8% each).

**Figure 2:**
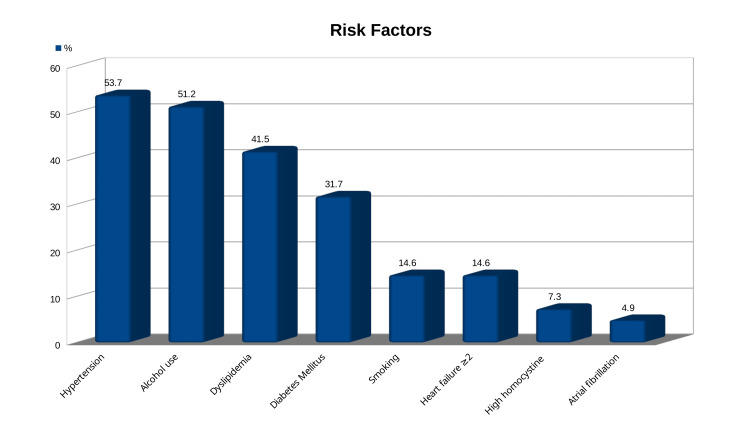
Risk factors associated with lateral medullary syndrome

Out of 41 patients, 22 (53.7%) had hypertension, all of whom were on anti-hypertensive (at least two) medications. Most patients (21, 51.2%) were alcohol consumers, among whom 7 (17.05%) had a history of heavy alcohol intake. Dyslipidemia was found in 17 patients (41.15%), diabetes in 13 (31.7%), and 6 patients (14.4%) who had a history of smoking. 18 (43.9%) of the patients had systolic heart failure, with 12 (29.3%) having grade I, 4 (9.8%) having grade II, 1 (2.4%) having grade III, and 1 (2.4%) having grade IV, according to the New York Heart Association (NYHA). Atrial fibrillation was present in two patients (4.9%); three patients (7.3%) had high homocysteine levels, among which two were below 50 years of age (Figure 2).

Of the 41 patients, 36 had MRA locating the vessel, and in five patients, MRA was non-revealing. The mean hours from symptom/s onset to MRI was 74.44 ± 49.8 hours, with a minimum of 4 hours and a maximum of 171 hours. Of those 36 patients with significant MRA, 31 patients had LMS due to vertebral artery (VA) pathology, where 8 patients had proximal and 11 had distal VA involvement, and 8 patients had both proximal and distal VA, i.e., complete vessel involvement. Of those, nine patients had PICA infarction. Of the 36 patients, 28 had large vessel infarction and 8 patients had small vessel (lacunar) infarction.

## Discussion

Ischemic stroke accounts for 83% of all acute strokes, with 20% involving the posterior circulation [7]. The most common cause of LMS is occlusion of the intracranial portion of the vertebral artery (67%), followed by PICA and its branches (10%) [2]. We found a 5:1 male-to-female ratio aged from 25 to 86 years, with a predominance of LMS in their sixth decade, similar to the study by Kim and Lui et al. [2,7].

Here, we aimed to link clinical symptoms to MRI findings. Although it is difficult to define the various lesions precisely, we sought to split the cases into rostrocaudal and transverse sections, using their characteristics to highlight possible clinical differences [5]. We found differences in the symptoms and signs of patients classified radiologically, which shows that radiological classification could be considered while treating LMS patients.

In the rostrocaudal segment, rostral infarction was more common than the caudal, where the large subtype was commonest followed by dorsal and typical subtypes, which is comparable to a study conducted by Kim et al. In addition, the lateral subtype was common in the caudal region [5]. Unlike the study by Kim, ours did not find statistically significant results, and their rostral lesions tend to have a ventral subtype as the most prevalent [2].

In the transverse section, the most prevalent radiological classification was found to be large infarctions. The transverse section and rostrocaudal levels may be related to the anatomical course of vertebral arteries located adjacent to the lateral surface of the caudal medulla, which ascends rostrally on the ventral surface to fuse into the basilar artery at the pontomedullary junction [5].

We identified identical symptoms and signs similar to those in the study by Kim et al. of 33 individuals with LMS. We found headache, nausea/vomiting, swaying on the Romberg test, dizziness, lateropulsion on standing, diplopia, vertigo, hemibody or hemifacial sensory loss, dysarthria, and dysphagia as common symptoms of lateral medullary infarction. Likewise, Kim et al. also found that 12% of patients had mild contralateral hemiparesis, which is comparable to our finding (17.1%) [5].

Vestibulocerebellar signs and symptoms were found in almost all radiological subtypes, which is consistent with the study conducted by Searls et al. [8]. Even though hemisensory loss (contralateral body, ipsilateral face) is common in LMS, it was present in only one-fifth of the patients in our study [5,9]. Hemiparesis was seen in one-third of the patients in this study, although previous studies have shown it to be an uncommon presentation [10]. In less than 5% of patients, paroxysmal facial pain was limited to the ocular distribution of the trigeminal (V1) area [11].

Kim's study on LMS showed that patients with large subtype LMS had a higher prevalence of Horner's syndrome, gait ataxia, dysphagia, dysarthria, vertigo, facial paresis, and trigeminal neuropathy. In contrast, our study findings were different. Headaches, hemisensory loss, nausea/vomiting, swaying on the Romberg test, lateropulsion on standing, and dizziness. Prevalent ocular symptoms were diplopia and nystagmus. Headaches occurred most often in the ipsilateral occipital area, followed by the frontal region [2]. Likewise, dorsal infarcts in Kim’s study show prevalent ataxia, vertigo, Horner's syndrome, and dysphagia. However, this is not the case in our study, where headaches, nausea/vomiting, and dizziness were prevalent. Kim's study also found that Horner's syndrome, contralateral trigeminal sensory loss, dysphagia, and ataxia were more common in the ventral subtype, whereas swaying, dizziness, and dysarthria were more common in ours. Consistent with Kim's findings, ataxia and dysphagia were common in the typical subtype. Horner’s syndrome, vertigo, and hoarseness were also common in the same study, which were not present in our study; swaying, contralateral body loss of pain and temperature, and dysphasia. Instead of Horner's syndrome, dysphagia, and hoarseness, patients with lateral subtypes in our study had headaches, nausea/vomiting, swaying, ataxia, and dizziness.

Dorsal infarctions had the highest prevalence of altered level of consciousness, followed by lateral and large subtypes, most probably due to the involvement of the reticular formation passing through these anatomical landmarks, while patients with typical and ventral infarctions had an intact level of consciousness [12]. Nausea/vomiting was more common in the lateral, large, and dorsal subtypes than in the typical and ventral, likely due to the projection to the adjoining nucleus trasctus solitarius with irritation of the area postrema [9,13]. Nausea and vomiting are also attributed to lesions of the vestibular nuclei [14]. Akin to Kim's study, dizziness was more common in the ventral, dorsal, and large groups. Large infarcts also had more nystagmus and sensory symptoms like altered pain and temperature in the body and face. Hemiparesis and facial palsy were most common in large infarcts but were almost non-existent in ventral infarcts. Diplopia was observed in the large subtype, followed by lateral, typical, and dorsal. This could be explained by the involvement of the medial longitudinal fasciculus or vestibular nucleus; surprisingly, it was also noted in the lateral subtype [15]. Diplopia in medullary lesions is also due to a compromise of the otolith ocular pathways, causing skew deviation [16].

Contrary to Kim’s study, dysarthria was most prominent in the ventral type and was absent in typical infarcts, whereas it was intermittently present in others. Similar to the study by Kim, dysphagia was least present in the dorsal group compared with the typical type [2]. Trigeminal neuropathy was uncommon among the patients, which was only present in large and ventral infarcts. Large and lateral types exhibited more swaying on the Romberg test. Likewise, nystagmus and lateropulsion upon standing were evident, especially in the large type.

In the rostral part of the medulla, more severe headaches, changes in the level of consciousness, dysphagia, and gait ataxia were linked to lesions, while vertigo, nausea/vomiting, nystagmus, dysarthria, ataxia, hiccups, and hemiparesis were linked to lesions in the caudolateral part of the medulla, which is similar to Kim's study [2,5]. Horner's syndrome, hoarseness, and vertigo were all prevalent symptoms, regardless of the location of the lesion. Vertigo and nausea/vomiting were common due to the close proximity of the area postrema in the dorsal type. Additionally, the nucleus intercalatus was involved, which caused upbeat nystagmus, and the nucleus prepositus hypoglossi was implicated, which caused gaze-holding failure [17].

However, hemiparesis is uncommon in LMS. We discovered few in our study, which can be explained by the fact that it is described in extensive areas of involvement in the dorsoventral region of the caudal medulla. Contralateral hemiparesis can arise if the pyramidal tract is disrupted before decussation. Ipsilateral hemiparesis occurs when an infarct spreads caudally and extends to the bridging corticospinal fibers, which are fed by the medullary penetrating arteries that start from the distal vertebral artery or the anterior spinal artery [18-20]. Hemiparesis can also be caused by hemi-medullary syndrome, medial medullary syndrome, or involvement of the anterior spinal artery with lateral spinal cord infarction, which is uncommon in association with LMS [21,22].

A prospective cross-sectional study on ocular lateral deviation (OLD) conducted by Kattah et al. opened the scope for future prospective studies, where the author discusses that LMS is commonly associated with OLD. OLD is the horizontal deviation of the eye to the side of the lesion that is made more prominent by brief eye(3-5s) closure [23]. Authors have noted that OLD in imaging (RadOLD) was common among anterior circulation stroke and horizontal gaze palsy but also associated with posterior circulation strokes, such as LMS and cerebellar stroke, without clinical correlation with OLD [23].

In terms of risk factors, Lui et al. discovered that hypertension was the most prevalent risk factor in infarction-related LMS, followed by smoking and diabetes [7]. Furthermore, as Day et al. pointed out, we discovered that alcohol use and dyslipidemia, both of which contribute to the formation of atherosclerosis and embolism, had a substantial impact on stroke [4,24]. According to our findings, heart failure and atrial fibrillation both elevate the risk of thromboemboli and infarction. High homocysteine increases stroke risk, especially in hypertensives. In line with Bots et al., we also found that the level of homocysteine was higher in people under 50 than in people over 50 [25].

Limitations

The comparison of MRI with clinical findings always has an inherent limitation that the clinical findings evolve over time, and initial findings may be related to the ischemic penumbra and resolve. The physician's variation in the clinical examination can also change the finding. Therefore, some of the initial findings may not have an "imaging correlate." Because of the retrospective nature of our study, we could not extract the long-term follow-up symptoms of the patients in this study.

In this retrospective study, the data revealed that the patients were not examined for OLD, which if done at that time, could have provided meaningful data. We consider it a limitation of our study and suggest considering these factors in future prospective studies. The sample size for this single-center-based study was not significantly large, implying that the study may not be applicable in all cases.

## Conclusions

LMS can be classified horizontally and rostrocaudally into various groups. The prevalence of symptoms in LMS has been well studied. However, the prevalence of symptoms based on radiological classification and individual circuit elements remains unclear, and still, LMS is a poorly studied topic. We found differences in the symptoms and signs of patients classified radiologically, which shows that radiological classification could be considered while treating LMS patients. We have tried to explain symptoms and signs in relation to the track, reticular activation system, and nucleus affected in either type of LMS, reemphasizing the anatomical consideration to be made for accurate localization of lesions in brainstem pathology. Our study will urge other researchers to conduct studies on various aspects of radiologic subtypes of LMS. This study may help in the development of treatments to prevent complications and predict the outcome.
